# Specific Strains of Lactic Acid Bacteria Differentially Modulate the Profile of Adipokines *In Vitro*

**DOI:** 10.3389/fimmu.2017.00266

**Published:** 2017-03-13

**Authors:** Emanuel Fabersani, María Claudia Abeijon-Mukdsi, Romina Ross, Roxana Medina, Silvia González, Paola Gauffin-Cano

**Affiliations:** ^1^Centro de Referencia para Lactobacilos (CERELA) – CONICET, Tucumán, Argentina; ^2^Facultad Ciencias de la Salud, Universidad del Norte Santo Tomás de Aquino (UNSTA), Tucumán, Argentina; ^3^Universidad Nacional de Tucumán (UNT), Tucumán, Argentina

**Keywords:** adipokine, leptin, lactic acid bacteria, probiotics, macrophage, adipocyte

## Abstract

Obesity induces local/systemic inflammation accompanied by increases in macrophage infiltration into adipose tissue and production of inflammatory cytokines, chemokines, and hormones. Previous studies have shown that probiotics could improve the intestinal dysbiosis induced by metabolic diseases such as obesity, diabetes, and metabolic syndrome. Microorganisms could (directly or indirectly) affect adipokine levels due to their capacity to induce translocation of several intestinal microbial antigens into systemic circulation, which could lead to metabolic endotoxemia or produce immunomodulation in different organs. The aim of the present study was to select non-inflammatory lactic acid bacteria (LAB) strains with the capacity to modulate adipokine secretion by the adipose tissue. We wish to elucidate the role of potential probiotic strains in the regulation of the cross talking between immune cells such as macrophages and adipose cells. Mouse macrophage cell line RAW 264.7 was used for evaluating the ability of 14 LAB strains to induce cytokine production. The LAB strains were chosen based on their previously studied beneficial properties in health. Then, in murine adipocyte culture and macrophage–adipocyte coculture, we determined the ability of these strains to induce cytokines and leptin secretion. Tumor necrosis factor alpha, interleukin 6 (IL-6), IL-10, monocyte chemoattractant protein-1, and leptin levels were measured in cell supernatants. We also performed the detection and quantification of leptin receptor (Ob-Rb) expression in macrophage cell lines stimulated by these LAB strains. Differential secretion profile of cytokines in macrophage cells induced by LAB strains was observed. Also, the levels of Ob-Rb expression diverged among different LAB strains. In LAB-stimulated coculture cells (adipocytes and macrophages), we observed differential production of leptin and cytokines. Furthermore, we detected lower production levels in single culture than cocultured cells. The principal component analysis showed an association between the four clusters of strains established according to their inflammatory profiles and leptin adipocyte production and leptin receptor expression in macrophages. We conclude that coculture is the most appropriate system for selecting strains with the ability to modulate adipokine secretion. The use of microorganisms with low and medium inflammatory properties and ability to modulate leptin levels could be a strategy for the treatment of some metabolic diseases associated with dysregulation of immune response.

## Introduction

Since the discovery of leptin, 21 years ago (1), the adipose tissue was the focus of several functional studies, in such a way that nowadays it could be considered as a potent endocrine and immune organ (2–5). This feature is due to the peculiarity of the different cells that form this tissue: adipocytes, preadipocytes, endothelial cells, fibroblasts, and numerous immune cells including macrophages, B cells, T cells, and neutrophils mainly. The cross talk between these cells affects the composition of the adipose tissue-produced molecules (6, 7). By means of these molecules, called adipokines, the adipose tissue influences the regulation of several important physiological functions such as appetite, satiety, energy expenditure, insulin sensitivity and secretion, glucose and lipid metabolism, fat distribution, endothelial function, blood pressure, neuroendocrine regulation, and immune function. Under conditions of adipose tissue dysfunction, frequently associated with obesity or undernourishment, the secretion of adipokines is dysregulated, affecting physiological functions and the host’s health (8–13). Currently, more than 600 adipokines are known. The most studied ones include leptin, adiponectin, resistin, visfatin, plasminogen activator inhibitor-1, tissue factor, tumor necrosis factor alpha (TNF-α), transforming growth factor beta, monocyte chemoattractant protein-1 (MCP-1), interleukin 6 (IL-6), and IL-8 (9). Leptin and adiponectin are adipose-derived factors with particular interest due to their potential as therapeutics in obesity treatment (14). Syndromes of malnutrition associated with excess/lack or dysfunction of adipose tissue certainly belong to these kinds of diseases (10).

Previous studies showed that adipokine levels could be affected by changes in the gut structure or in the composition of microbiota (13). Scientific evidence from both animal and human studies supports the relationship between the composition and function of intestinal microbiota and the alterations observed in metabolic and inflammatory syndromes. Consequently, the design of more effective dietary strategies for improving the treatment and prevention of metabolic diseases *via* modification of the gut microbiota composition or function is of increasing interest (15). In this context, Cani et al. (16) reported that changes in gut microbiota (following a high-fat diet or genetically induced obesity) contribute to increased gut permeability, metabolic endotoxemia [higher serum lipopolysaccharide (LPS) levels], and low-grade inflammation-induced metabolic disorders (insulin resistance and diabetes), while its restoration by prebiotic and probiotic strategies ameliorate those adverse effects. Potential probiotic strains have been shown to play a role in nutritional disorders, including both under and overnutrition in human and animal studies (17–25).

In this context, we demonstrated that oral administration of potential probiotic bacteria to a high-fat diet-induced obesity mice model ameliorates the alterations of leptin and cytokine levels along with partial restoration of the obesity-induced dysbiosis (21, 22). Recently, *in vitro* and *in vivo* studies demonstrated the influence of lactobacilli and bifidobacteria on adipokine levels (18, 21, 26). Considering the pleiotropic role of leptin in energy balance, the administration of commensal bacteria able to regulate its secretion and function could be an attractive strategy for the management of malnutrition (undernutrition and obesity), but appropriate strains need to be selected (27).

Circulating levels of leptin, an adipocyte-secreted hormone, are decreased or increased in undernutrition or obesity, respectively, and have thus been proposed to be a link between nutritional status and immune function (28–30). Papathanassoglou et al. (31) suggested the notion that nutritional status, acting via leptin-dependent mechanisms, may alter the nature and vigor of the immune response. Moreover, these authors showed that Ob-Rb, the leptin receptor, is expressed in normal mouse lymphocyte subsets and that leptin plays a role in lymphocyte survival, which alters the Ob-Rb/STAT-3-mediated signaling in T cells (31). In addition, it has been suggested that microbial products associated with immunobiotics (immunoregulatory probiotics) can also cross the intestinal barrier, being able to modulate distant tissues (such as adipose tissue) through the induction of changes in cytokine profiles (32). Miyazawa et al. in an *in vitro* study using conditioned medium showed that different lactobacilli may suppress differentiation of preadipocytes through macrophage activation and alter the immune responses of macrophages to adipose cells (33).

Therefore, we consider significant to establish a suitable criterion for the selection of immunobiotic strains with anti-inflammatory properties and ability to modulate leptin production. These bacterial capacities in this “next-generation of probiotics” would be useful to be applied in alternative treatments for diseases in which the profile of adipokines is deregulated (obesity, metabolic syndrome) (27).

The aim of the present study was to select non-inflammatory lactic acid bacteria (LAB) strains with capacity to modulate adipokine secretion in the adipose tissue. The LAB strains were selected for this study by their probiotic properties previously determined (17, 23, 34–38). To achieve this purpose, first we evaluated in murine macrophages the ability of different strains to induce the secretion of cytokines and chemokines (pro-inflammatory and anti-inflammatory responses) involved in metabolic inflammation (TNF-α, IL-6, IL-10, and MCP-1). The infiltration of adipose tissue by macrophages is an important event causing increased inflammatory process in obesity (39–41). In addition, leptin and cytokines and one chemokine secretion in murine adipocytes (mono- and coculture with macrophages) were investigated in order to understand the role of potential probiotic strains in the adipokine modulation. We also evaluated the leptin receptor (Ob-Rb) expression in macrophages, a molecule constitutively expressed in immune cells such as macrophages, B cells, and T cells (31).

## Materials and Methods

### Bacterial Strains and Culture Conditions

Lactic acid bacteria strains were obtained from the Centro de Referencia para Lactobacilos (CERELA, Tucumán, Argentina) culture collection. They were isolated in our laboratory from regional products and stools of healthy infants. All the employed bacteria used in this work could potentially be considered as probiotic strains or as starter cultures for meat or milk fermentation. Probiotic properties previously determined were immunomodulation, production of conjugated linoleic acid, feruloyl esterase activity, protection against intestinal infections, etc (17, 23, 34–38).

Strains used in this study were *Lactobacillus casei* CRL431, *L. acidophilus* CRL258, *L. acidophilus* CRL1063, *L. casei* CRL66, *L. casei* CRL72, *L. casei* CRL117, *L. fermentum* CRL1446, *Lactococcus lactis* CRL1434, *L. plantarum* CRL350, *L. plantarum* CRL352, *L. plantarum* CRL353, *L. plantarum* CRL355, *L. paracasei* CRL575, and *L. rhamnosus* CRL576.

Bacteria were cultured in Man–Rogosa–Sharpe (MRS) broth (Britania, Buenos Aires, Argentina) at 37°C for 22 h (stationary growth phase). Cells were harvested by centrifugation (10,000 rpm for 10 min), washed twice with phosphate-buffered saline (PBS, 130 mM sodium chloride, 10 mM sodium phosphate, pH 7.4), and re-suspended in PBS containing 20% (v/v) glycerol. Aliquots of these suspensions were frozen and stored at −80°C until used for *in vitro* stimulation of macrophage and adipocyte cells. The number of viable cells after freezing and thawing was determined by colony-forming unit counting on MRS agar after 48 h incubation in microaerophilia. For each strain tested, more than 90% cells remained alive upon thawing, and no significant differences were found during storage time (2 months). One fresh aliquot was thawed for every new experiment to avoid variability in the viability of cultures in the experiments.

### Ability of LAB Strains to Induce Cytokine Secretion by Macrophages

Mouse macrophage cell line RAW 264.7 was used for evaluating the ability of different LAB strains to induce cytokine production as previously determined (21). Cells were cultured overnight into 24-well flat-bottom polystyrene microtiter plates (Costar^®^ 24 Well Clear TC-Treated Multiple Well Plates, Individually Wrapped, Sterile #3524) at a concentration of 1 × 10^5^ cells per milliliter in Dulbecco’s Modified Eagle’s Medium (DMEM—Gibco #41965-039). Medium was changed before stimulation, and then cells were incubated in the presence of 100 μl of a cell suspension (1 × 10^7^ CFU/ml) of each strain for 24 h. Purified LPS from *Escherichia coli* serotype O26: B6 (Sigma Chemical Co, USA, #L2654) was used as a positive control, at a final concentration of 1 μg/ml. Cell viability was assessed by the Trypan blue assay. Non-stimulated Raw 264.7 cells were also evaluated as controls of basal cytokine production (control negative). The cell culture supernatants were collected, centrifuged (10,000 rpm for 10 min), and stored at −20°C until cytokine determination.

### Detection and Quantification of Obesity Receptor (Ob-Rb) Expression

Macrophages were collected by vigorous pipetting after stimulation with different bacterial strains and immediately washed twice with PBS. Cells (10^6^) were incubated with primary polyclonal antibody rabbit antimouse leptin receptor (Ob-R H300: sc8325, Santa Cruz Biotechnology, Inc.) in PBS-bovine serum albumin (1% w/v), dilution 1/250 (v/v) for 1 h at room temperature, washed twice with PBS-BSA (5%, w/v), and incubated with FITC-conjugated secondary antibodies goat antirabbit IgG-FITC (IgG-FITC: sc-2012 Santa Cruz Biotechnology, Inc.) for an additional 1 h at room temperature in darkness. Cells were washed twice and resuspended in 500 μl PBS (1%, w/v). LPS-stimulated and non-stimulated macrophages were used as positive and negative controls of the leptin receptor expression. Macrophage Ob-Rb (+) was quantified using flow cytometry (BD/FACSCalibur). Gates were set to exclude cell debris and non-specific Ab binding, and results were analyzed using the FACS analysis software (CellQuest; BD Biosciences). Unstained controls were used for setting compensation and gates.

Immunofluorescence microscopy was used to detect the Ob-Rb leptin receptor in the macrophages. The cells were cultured overnight into 24-well flat-bottom polystyrene microtiter plates [Costar^®^ 24 Well Clear TC-Treated Multiple Well Plates, Individually Wrapped, Sterile (Product #3524)] containing glass coverslips 12 mm in diameter, at a concentration of 10^5^ cells per milliliter in DMEM, then, the cells were washed twice with PBS 1× and fixed with paraformaldehyde 1% (SIGMA P6148-500G) in PBS, and incubated with primary and secondary antibodies as previously described for flow cytometry. Finally, a drop of mounting medium (SIGMA F4680, USA) was placed on the labeled cells and then the coverslip, following visualization in a fluorescence microscope (Carl Zeiss—Axio Scope.A1).

### Isolation of Murine Adipocytes

Adipocytes from epididymal adipose tissue were isolated from 6-week old Balb/c male mice by collagenase digestion (collagenase from *Clostridium histolyticum* Type II #C6885 SIGMA, USA) as previously described (42, 43). Briefly, adipose tissues from four to six mice were pooled for adipocyte isolation. Tissue was digested during an hour incubation period at 37°C in DMEM high glucose supplemented with 10% (v/v) fetal bovine serum (FBS), penicillin/streptomycin, and containing 1.5 mg/ml collagenase II under constant agitation. The suspension was subsequently centrifuged at 500 rpm for 5 min at room temperature. The mature adipocyte floated to the surface, and the stromal vascular cells (capillary, endothelial, mast, macrophage, and epithelial cells) were deposited at the bottom. The stromal vascular cells were removed by aspiration, and the fat cells were washed with 10 ml of DMEM high glucose supplemented with 10% FBS and penicillin/streptomycin and centrifuged at 400 rpm for 2 min. This procedure was repeated twice. Two aliquots (10 μl) of cell suspension were stained with Trypan blue and counted on a hemocytometer to estimate the concentration of adipocyte cells. The experimental protocol was approved by the Animal Protection Committee of CERELA (CRL-BIOT-EF-2012/2A) and complied with current Argentinean laws.

### Ability of LAB Strains to Induce Cytokine and Leptin Secretion by Murine Adipocytes Culture and Macrophage-Adipocyte Coculture

#### Adipocyte Culture

Adipocyte cells in 0.5 mL of plating medium were inoculated into 24-well plates at a concentration of 5 × 10^5^ cells/wells, to study the ability of different strains to induce leptin and cytokine production. The cells were maintained in a humidified 5% CO_2_ atmosphere for 24 h at 37°C. Medium was changed before the LAB stimulation, and then adipocytes were incubated in the presence of 100 μl of a cell suspension 1 × 10^7^ CFU/ml of each strain for 24 h. Purified LPS from *E. coli* serotype O26:B6 was used at a concentration of 1 μg/ml as a positive control. Non-stimulated adipocytes cells were also evaluated as controls of basal leptin and cytokine production (control negative). Cell viability was assessed by the Trypan blue assay.

#### Macrophage–Adipocyte Coculture

Experiments of murine adipocytes and macrophages (cell line RAW 264.7) were performed by triplicate in two independent experiments with each strain. Adipocytes were seeded at a concentration of 10^4^ cells/ml into 24-well plates, and macrophage cells were previously cultured at a concentration of 10^4^ cells/ml. The cells were cocultured in a humidified 5% CO_2_ atmosphere for 24 h at 37°C. Media were changed before stimulation, and then cells were incubated in the presence of 100 μl of each strain suspension (1 × 10^7^ CFU/ml) during 24 h at 37°C in a humidified 5% CO_2_ atmosphere. Purified LPS from *E. coli* serotype O26:B6 was used at a concentration of 1 μg/ml as a positive control. Non-stimulated coculture cells were also evaluated as controls of basal leptin and cytokine production (control negative). The coculture supernatants were collected and stored at −20°C until cytokines, chemokine, and leptin were quantified.

#### Detection of Cytokines and Adipokines

Tumor necrosis factor alpha, IL-6, MCP-1, and IL-10 were quantified in cell supernatants using the BD™ Cytometric Bead Array Mouse Inflammation Kit (BD Bioscience, 560485, San Diego, CA, USA). Leptin concentrations were measured in cell supernatants with a DuoSet kit (R&D Systems, Minneapolis). The assays were performed according to the manufacturer’s instructions.

### Statistical Analyses

Statistical analyses were performed using SPSS version 17.0 software (SPSS Inc., Chicago, IL, USA). The data were normally distributed and significant differences were determined by applying one-way ANOVA with *post hoc* Tukey’s test or Fisher’s least significant difference test. In every case, *P*-values < 0.05 were considered statistically significant. Principal component analysis (PCA), as a descriptive/exploratory technique, was used in order to reveal relationships between the variables. Further interpretations of any relationships among strains, cytokine and leptin levels, and Ob-Rb expression were obtained using PCA and Pearson correlation coefficients. PCA analysis was performed using the software XLSTAT (18.06).

## Results

### Differential Secretion Pattern of Cytokines/Chemokine Induced by Different LAB in Macrophages

**Figure 1:**
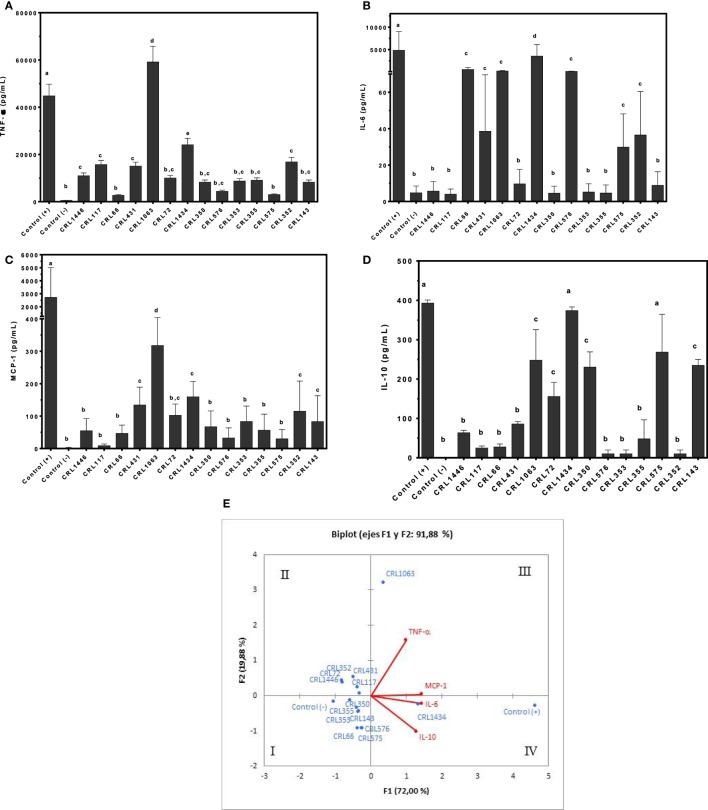
**Production of tumor necrosis factor alpha (TNF-α), interleukin 6 (IL-6), monocyte chemoattractant protein-1 (MCP-1), and IL-10 by RAW 264.7 cells stimulated with different lactic acid bacteria (LAB) strains**. Cytokines/chemokine and leptin concentrations were assayed in supernatants from LAB-stimulated RAW 264.7 cells. Macrophages were treated for 24 h with bacterial cell suspensions, and TNF-α **(A)**, IL-6 **(B)**, MCP-1 **(C)**, and IL-10 **(D)** levels were quantified using cytometric bead array kit and leptin levels, using enzyme-linked immunosorbent assay test. Purified lipopolysaccharide from *Escherichia coli* was used as a positive control. Non-stimulated cells were evaluated as controls of basal cytokine levels (negative control). The stimulating strains were CRL431, CRL258, CRL1063, CRL66, CRL72, CRL117, CRL1446 CRL1434, CRL350, CRL352, CRL353, CRL355, CRL575, and *L*. CRL576 strains. Results are expressed as mean ± SD of triplicate measures determined in two independent experiments. Mean values in the same graphic with different letters (a–d) were significantly different (*P* < 0.05). The relationships among strains, cytokines/chemokine wer obtained using (PCA) and Pearson correlation coefficients. In the biplot obtained by PCA **(E)**, points are coded by stimulating strains and controls. The position of some points was slightly modified to avoid overlapping of the labels.

Results in Figure 1 show the ability of viable LAB to induce cytokines/chemokine production in macrophages. Production of TNF-α and IL-6 (pro-inflammatory cytokines), IL-10 (regulatory cytokine), and MCP-1 (pro-inflammatory chemokine) by macrophages stimulated with several strains was determined. Large differences in the inflammatory profile among surveyed strains were observed. These results showed that the inflammatory property is strain-dependent. All strains induced lower secretion of pro-inflammatory and regulatory cytokines and chemokine than positive control.

Tumor necrosis factor alpha showed values lower than LPS stimulus (positive control), with exception of CRL1063 strains. CRL66 and CRL575 produced the lowest TNF-α secretions (Figure 1A). The highest secretion of IL-6 was observed when the cells were stimulated with strain CRL1434. Intermediate values of IL-6 were observed with CRL66, CRL431, CRL1063, CRL576, CRL575, and CRL372 strains, while with CRL1446, CRL117, CRL72, CRL350, CRL353, CRL355, and CRL143 strains, the lowest values of IL-6 were obtained (Figure 1B). In addition, CRL 1063 induced the highest production of MCP-1 followed by CRL431, CRL72, CRL352, and CRL143 (Figure 1C). Regarding anti-inflammatory cytokine IL-10, the highest levels were obtained in cells stimulated by strains CRL1434 and CRL575, followed by strains CRL1063, CRL72, CRL350, and CRL143 (Figure 1D).

The PCA (Figure 1E) revealed an association between strains into four different groups, according to the inflammatory mediators induced. The cluster I included the CRL355, CRL353, CRL350, CRL576, CRL66, and CRL575 strains. This cluster also comprises the negative control that represents the baseline cytokine secretion, which is positioned far from other strains with a lower inflammatory profile. The cluster II included the CRL117, CRL1446, CRL72, CRL431, and CRL352 strains and in the cluster III, CRL1063 was very closely located in the limit between quadrants II and III. The cluster IV only included the strain CRL1434 with a high inflammatory profile next to LPS stimulus (positive control), since this stimulus produced the highest inflammatory cytokine secretion compared to all studied strains.

### Expression of the Ob-Rb on Macrophage RAW 264.7

**Figure 2:**
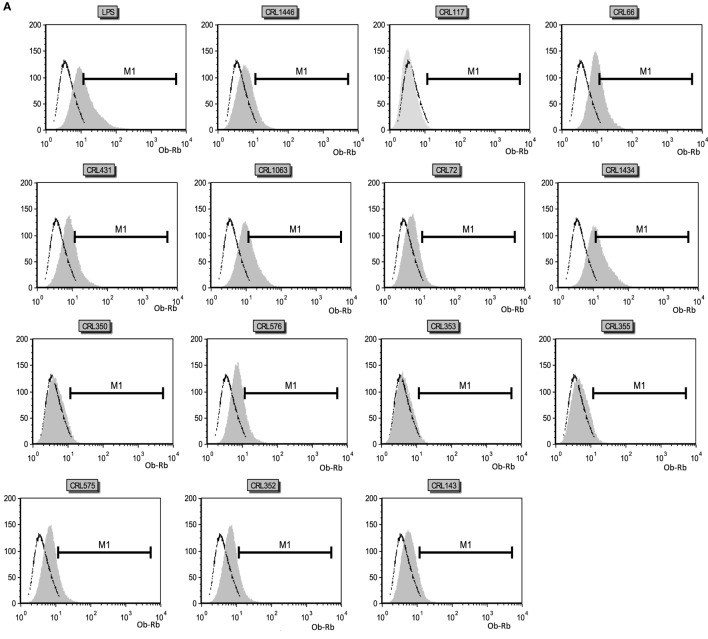

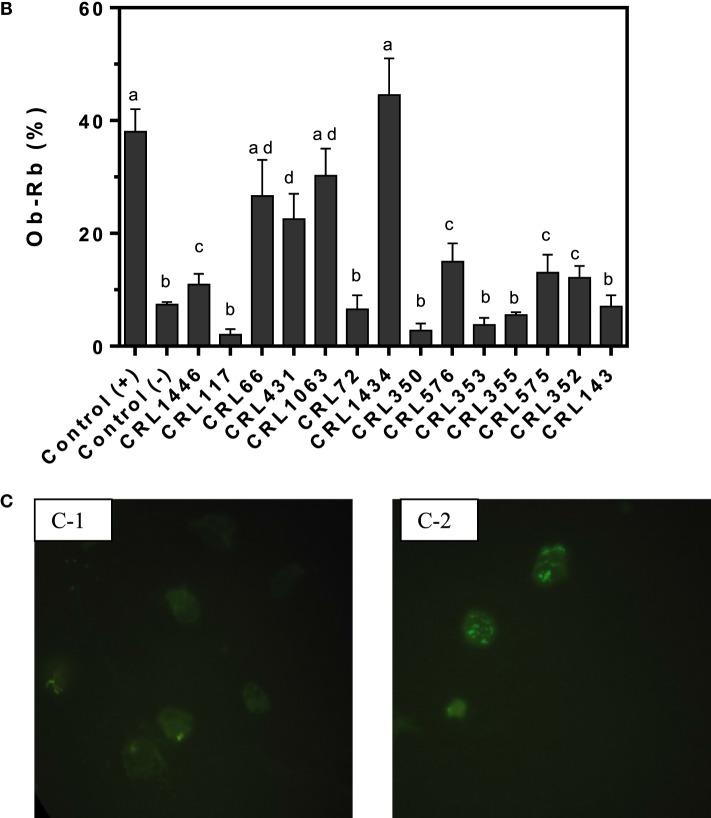
**Leptin receptor (Ob-Rb) expression in RAW 264.7 cells**. RAW 264.7 cells after lactic acid bacteria (LAB) stimulation were incubated with a polyclonal rabbit antimouse Ob-Rb Ab and a secondary FITC-conjugated Ab (antirabbit IgG). Ten thousand cells were analyzed per sample at an argon laser flow cytometry scanner. **(A)** Histograms correspond to the analysis of Ob-Rb expression by non-stimulated (negative control) and LAB-stimulated RAW 264.7 cells. The stimulating strains were CRL431, CRL258, CRL1063, CRL66, CRL72, CRL117, CRL1446 CRL1434, CRL350, CRL352, CRL353, CRL355, CRL575, and CRL576. Lipopolysaccharide (LPS) was used as a positive control. Open black line represents fluorescent labeling of Ob-Rb positive cells of negative control, and gray areas represent other strains stimulus. **(B)** Percentage of Ob-Rb expression in RAW 264.7 cells. The bars represent the different LAB strains. The results are expressed as media of percentages of cells Ob-Rb (+) ± SD of two experiments. Mean values in the same graphic with different letters (a–d) were significantly different (*P* < 0.05). **(C)** Microphotograph representative of the Ob-Rb expression in non-stimulated macrophages (negative control, C-1) and LPS-stimulated macrophages culture (positive control, C-2).

The influence of different LAB strains on Ob-Rb (leptin receptor) expression in macrophages is shown in Figure 2. Basal Ob-Rb expression was detected in approximately 7.5% of control macrophages (10^6^ cells; negative control or non-stimulated cells; Figures 2A,B). Cells were subsequently stimulated during 24 h by either different LAB or LPS (control positive). Ob-Rb-positive cells increased to a maximum value of about 38% after LPS stimulation, while the percentages of LAB-stimulated macrophages positive for Ob-Rb were lower, except for CRL1434. Levels of Ob-Rb expression diverged among different LAB stimuli. The most significant increase in the percentage of Ob-Rb (+) cells was observed when strain CRL1434 was used as stimulus. The strains CRL66 and CRL1063 induced similar Ob-Rb expression to positive control. CRL1446, CRL431, CRL576, CRL575, and CRL143 strains increased the positive macrophages compared to the basal control. For the other studied LAB strains, no significant changes in Ob-Rb expression were observed compared to negative control. The qualitative immunohistochemistry analyses were also performed in macrophages (Figure 2C).

### Differential Production of and Cytokines/Chemokine and Leptin in Adipocytes Stimulated with Different LAB Strains

**Figure 3:**
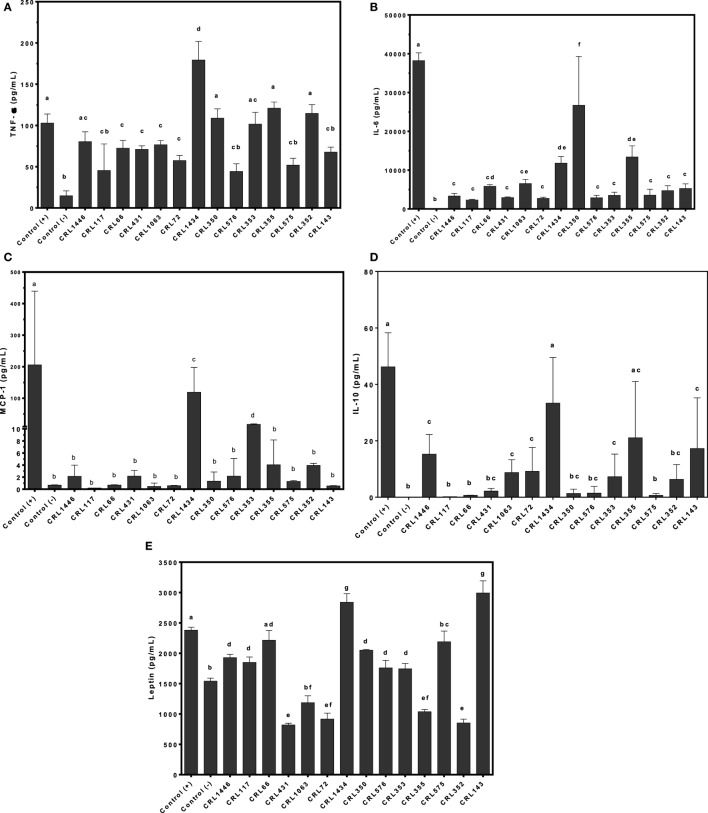
**Production of TNF-α (A), interleukin 6 (IL-6) (B), monocyte chemoattractant protein-1 (MCP-1) (C), IL-10 (D), and leptin (E) by lactic acid bacteria (LAB)-stimulated mice adipocytes**. Cytokines/chemokine and leptin concentrations were assayed in supernatants from LAB-stimulated mice adipocytes. Adipocytes were treated for 24 h with bacterial cell suspensions, and TNF-α, IL-6, MCP-1, and IL-10 levels were quantified using Cytometric Bead Array Kit and leptin levels, using enzyme-linked immunosorbent assay test. LPS was used as a positive control. Non-stimulated cells were evaluated as controls of basal cytokine levels (negative control). The stimulating strains were CRL431, CRL258, CRL1063, CRL66, CRL72, CRL117, CRL1446 CRL1434, CRL350, CRL352, CRL353, CRL355, CRL575, and *L*. CRL576 strains. Results are expressed as mean ± SD of triplicate measures determined in two independent experiments. Mean values in the same graphic with different letters (a–g) were significantly different (*P* < 0.05).

To evaluate whether LAB may directly induce secretion of leptin, cytokines, and chemokine in adipocytes, we stimulated murine adipocyte cultures with several strains. We observed different effects of LAB on TNF-α, IL-6, IL-10, and MCP-1 adipocyte production. The CRL1434 strain induced the highest values of TNF-α; MCP-1 and IL-10 compared to the other strains (Figures 3A,C,D), while CRL350 strain induced the highest levels of IL-6 (Figure 3B).

As shown in Figure 3E, leptin production is modulated by LAB in a strain-dependent manner. We showed that LPS was able to induce a significantly higher leptin production compared to negative control. Similar effect was observed for CRL66 and CRL575 strains. The CRL1434 and CRL143 strains induced more leptin production than positive control. In contrast, lower levels of leptin secretion were secreted by adipocytes treated with the CRL431, CRL1063, CRL72, CRL355, and CRL352 strains compared to negative control. Leptin levels produced by adipocyte stimulated with CRL1446, CRL117, CRL350, CRL576, and CRL353 strains were significantly from those detected in the negative and positive controls.

### Differential Production of Cytokines/Chemokine and Leptin by Cocultured Cells (Adipocytes and Macrophages) Stimulated with Different LAB Strains

**Figure 4:**
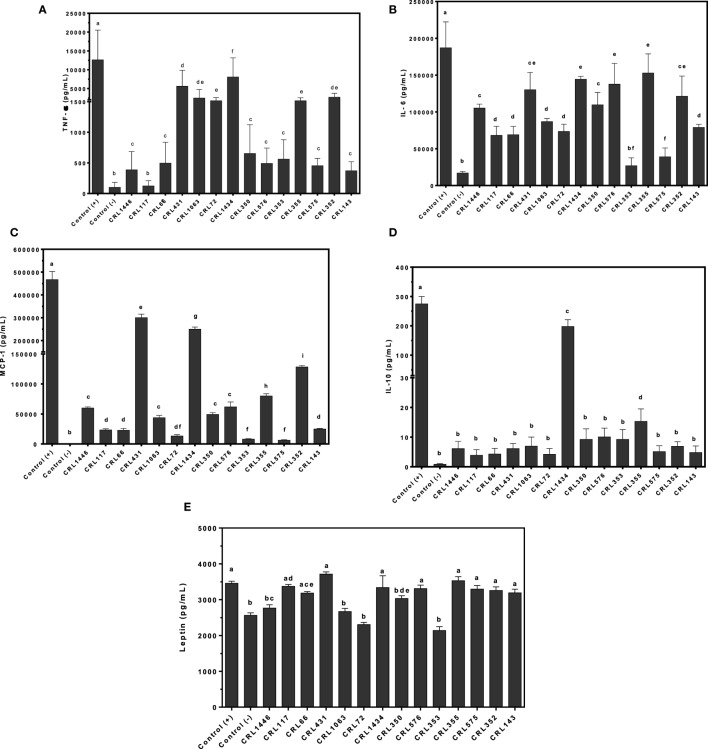
**Production of tumor necrosis factor alpha (TNF-α), interleukin 6 (IL-6), monocyte chemoattractant protein-1 (MCP-1), IL-10, and leptin by macrophage/adipocyte coculture stimulated with lactic acid bacteria (LAB) strains**. Cytokines/chemokine and leptin concentrations were assayed in supernatants from LAB-stimulated macrophage/adipocyte coculture. Cells were treated for 24 h with bacterial cell suspensions and TNF-α, IL-6, MCP-1, and IL-10 levels were quantified using Cytometric Bead Array Kit and leptin levels, using enzyme-linked immunosorbent assay test. LPS was used as a positive control. Non-stimulated cells were evaluated as controls of basal cytokine levels (negative control). The stimulating strains were CRL431, CRL258, CRL1063, CRL66, CRL72, CRL117, CRL1446 CRL1434, CRL350, CRL352, CRL353, CRL355, CRL575, and *L*. CRL576 strains. Results are expressed as mean ± SD of triplicate measures determined in two independent experiments. Mean values in the same graphic with different letters (a–i) were significantly different (*P* < 0.05). Production levels of **(A)** TNF-α, **(B)** IL-6, **(C)** MCP-1, **(D)** IL-10, and **(E)** leptin.

To assess whether LAB may directly induce production of TNF-α, IL-6, MCP-1, IL-10, and leptin by the macrophage–adipocyte coculture, we stimulated these cells with the bacterial strains (Figure 4). Different effects of LAB on cytokine production in the cell coculture were observed (Figures 4A–D). All strains induced lower cytokines production than positive control. The CRL1434 strain was the most effective stimulating both inflammatory (TNF-α, IL-6, and MCP-1) and anti-inflammatory (IL-10) cytokines secretion. The CRL431, CRL1063, CRL72, CRL355, and CRL352 strains induced significantly higher TNF-α values than CRL1446, CRL66, CRL350, CRL576, CRL353, CRL575, and CRL143 (Figure 4A). Regarding IL-6 production, almost all strains induced intermediated levels between positive and negative controls (Figure 4B). The CRL431 strain was the most potent inductor of MCP-1 secretion (Figure 4C), meanwhile CRL1434 strain was the best inductor of IL-10 secretion (Figure 4D).

Leptin production was also modulated by LAB in a strain-dependent manner. The CRL17, CRL66, CRL431, CRL1434, CRL576, CRL355, CRL575, CRL352, and CRL143 strains induced similar levels of leptin than positive control. Other group of strains (CRL1446 and CRL350) induced mid-levels of leptin. The CRL1063, CRL72, and CRL353 strains did not induce significant changes in leptin production compared with negative control.

### Associations between Strain and Inflammatory Profile, Expression of the Ob-Rb (Macrophage), and Leptin Production (Adipocyte)

**Figure 5:**
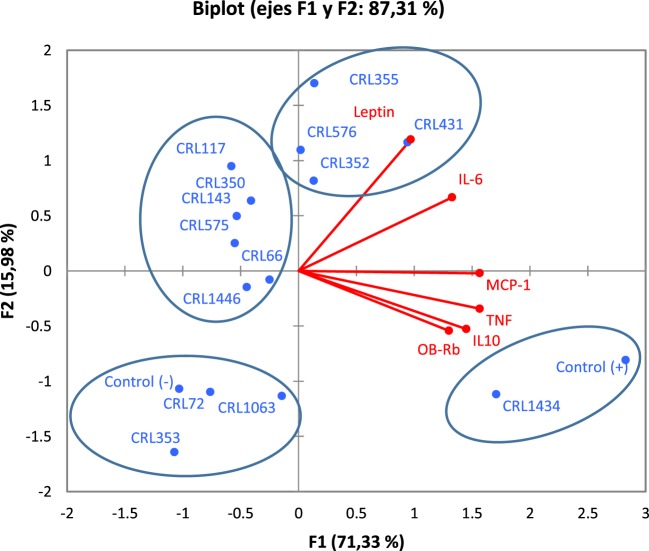
**Biplots obtained by principal component analysis (PCA) of cytokines/chemokine and leptin production by macrophage/adipocyte coculture associated with expression of the Ob-Rb receptor in macrophages**. Levels of TNF-α, IL-6, MCP-1, IL-10, and leptin produced by macrophage/adipocyte cocultures were associated with the expression of Ob-Rb in macrophages in biplots obtained by PCA. Non-stimulated cells were evaluated as controls basal (negative control) of cytokines/chemokine and leptin levels and Ob-Rb expression. LPS was used as a positive control. In the biplots, points are coded by stimulating strains (CRL431, CRL258, CRL1063, CRL66, CRL72, CRL117, CRL1446 CRL1434, CRL350, CRL352, CRL353, CRL355, CRL575, and *L*. CRL576) and controls. The position of some points was slightly modified to avoid overlapping of the labels.

Figure 5 shows the PCA plots with focus on grouping of LAB with respect to cytokines and leptin produced by macrophage–adipocyte coculture, both associated with expression of the Ob-Rb in RAW 264.7 macrophages. PCA revealed the presence of different groups of LAB with significant biological correlation (Figure 5) according to the studied variables. The analysis clearly discriminated between LAB with different capacity for modulating adipokine production. The analysis revealed that two components can be extracted, which together accounted for 87.31% of the variability. However, the first component accounts for 71.33% of the variance and discriminated better the LAB according to their capacity for modulating adipokine production and was more influenced by TNF-α and MCP-1; however, all variables were positively correlated. Second component separates better the strains with regard to their influence on leptin and IL-6 levels. So, the first and second factors discriminate LAB strains in four clusters according to their inflammatory profiles, leptin (from adipocyte to macrophage) production and leptin receptor expression (only in macrophages).

In the cluster I, we observed strong correlations between the CRL72, CRL1063, and CRL353 strains. This group also comprises the negative control (basal control). The cluster II includes CRL1446, CRL66, CRL575, CRL143, CRL350, and CRL117 strains. The cluster III includes the CRL352, CRL576, CRL355, and CRL431 strains. The CRL1434 strain close to positive control (LPS control) was located in the cluster IV. Stimulus with CRL1434 strain induced the highest inflammatory cytokine levels compared to the other strains studied.

## Discussion

This study supports the hypothesis that LAB have capacity to induce adipokine secretion in strain-dependent manner, and this ability is related to their immune properties. This report also provides additional evidence for the role of leptin in the regulation of the immune system and the cytokine network. In addition, we showed a strategy of *in vitro* selection of immunobiotic strains (immunoregulatory probiotics) to prevent or improve immunometabolic alterations associated with malnutrition.

The mechanisms by which commensal bacterial strains (particularly lactobacilli and bifidobacteria) may confer health benefits on the host’s health have been extensively studied (15). LAB are known to exert a wide range of effects on the immune and endocrine systems and, therefore, potentially in the whole body metabolism. However, these effects may be strain-specific and properties of one strain cannot be extrapolated to another even if belonging to the same species (44). Several beneficial microorganisms, generally known as immunobiotics (38), have been investigated for their potential use to treat or prevent nutritional disorders (undernutrition and obesity), which are further associated with immune deregulation (17, 21, 38, 45, 46).

Obesity induces low-grade inflammation characterized by chronic elevations in circulating inflammatory cytokines and adipokines (47, 48). Adipose tissue is constituted by active endocrine cells that secret a number of adipokines, which play pivotal roles in the regulation of various physiological and pathological processes in which adipose tissue is involved (49). At the adipose tissue level, inflammatory pathways are induced due in part to dynamic quantitative and phenotypic changes in adipose tissue leukocytes, principally macrophages (48, 50). According to Weisberg et al. (39), in mouse models, macrophage content could increase to about 50% of total non-adipocyte cells in obese mice. Macrophages infiltrated in white adipose tissue are the principal source of IL-6 and TNF-α (51). TNF-α is a pro-inflammatory cytokine, which has also been implicated as a mediator in induction of insulin resistance and adipose tissue inflammation (52). IL-6 has long been regarded as a pro-inflammatory molecule, but recent findings suggest that it also has many anti-inflammatory effects (53). IL-10 is a strong anti-inflammatory cytokine due to its ability to suppress the release of TNF-α and IL-1b from macrophages. In addition, IL-10 would affect Th1-lymphocyte actions (54). Regarding this, it was suggested that the C-C chemokine receptor 2 and its ligand CCL2 or MCP-1 are necessary for accumulation of inflammatory macrophages (39).

The results showed in this work indicate that adipocyte–macrophage coculture could be used for the screening and the selection of new immunobiotic strains, with the potential to functionally modulate adipose inflammation when orally administered. On the other hand, several authors have demonstrated that the gut microbiota could be modified by oral administration of LAB, and it has been identified as an important modifier of systemic inflammatory reactions influencing remote tissues (21). In addition, it is important to mention that there is no direct contact between intestinal bacteria and adipose tissue, but this contact could be indirectly mediated through translocation of bacterial products (e.g., LPS, DNA, etc.), which reach peripheral tissues and can stimulate innate immune receptors such as the mammalian toll-like receptors (TLRs) (55, 56). TLRs are germ line-encoded receptors expressed by cells of the innate immune system, adipocytes or intestinal epithelia, which are stimulated by structural motifs characteristically expressed by bacteria, viruses, and fungi known as pathogen-associated molecular patterns (57). Food-derived fatty acids, as well as intestinal bacteria-derived fatty acids could be sensed by TLRs, resulting in activation of the immune system (55). Taking this into account, we and other authors suggested that disruption of the mucosal barrier, as occurs in obesity and or undernutrition, leads to the exposure of a multitude of commensal-derived TLR ligands that could interact with TLR-expressing immune cells and adipocytes (55, 56). Batra et al. (58) demonstrated that expression and responsiveness of TLRs (TLR 1–9) in murine preadipocytes and adipocytes are both strongly regulated by leptin. This, coupled with the demonstrated pro-inflammatory actions of leptin in a variety of immune cells and other cell types (59), led us to propose that inflammatory responses could be regulated by leptin-modulating strains.

Adipose tissue consists of active endocrine cells that secrete a large number of adipokines, mainly leptin, adiponectin, and inflammatory cytokines such as TNF-α, IL-6, IL-1, IL-10, and MCP-1. These factors play a key role in the regulation of various physiological and pathological processes in which adipose tissue is involved (49, 60). For this purpose, we evaluated the ability of LAB to induce changes in the secretion of adipokines, cytokines, and chemokines involved in metabolic inflammation (IL-6, IL-10, TNF-α, MCP-1, and leptin), macrophages and adipocytes. Main cells that compose the adipose tissue (6, 61).

In order to establish the influence of cross talk between the main cells of adipose tissue, a coculture technique using macrophages and adipocytes was developed. According to Aravindhan and Madhumitha, metainflammation is due to the dysfunction of the immune system: at optimal level it confers protection against pathogens; at the suboptimal level it leads to immunodeficiency; and at supraoptimal level it leads to inflammation (62). Taking this into account, it is necessary to know the inflammatory profile of potential probiotic LAB. In this study, we first examined the ability of LAB to induce the production of different pro-inflammatory and anti-inflammatory responses in murine macrophage cell line, since the high infiltration of adipose tissue by macrophages occurs in obesity (39–41). Macrophages are phagocytic cells that participate in the innate and adaptive immune system. They are also one of the major cells that are in contact with the microorganisms in the intestine (63). This contact can be performed by phagocytosis or by recognition through different receptors, such as those PMAMs, including TLRs. In addition to the intestine, macrophages are found in various tissues, being one of the main immune cells of the visceral adipose tissue, in both lean and obese subjects, where they can represent between 5 and 60% of the immune cells of the adipose tissue (64, 65).

In the metabolic disorders, the number and level of activation of macrophages are altered (66), so that metabolic parameters, such as insulin resistance associated, may be affected (67) or the response to infections associated with malnutrition (68, 69). The main reason is due to the level of dynamic polarization between classically activated macrophages M1 (producers of pro-inflammatory cytokines) and alternatively activated macrophages M2 (anti-inflammatory cytokine producers) present in the adipose tissue (6). The level of activation is associated with a particular cytokine profile, and this is dysregulated in metabolic disorders (70). We demonstrated large differences in the inflammatory profile among evaluated strains, suggesting that the inflammatory property is strain-dependent, and different functional roles could be played by studied strains. We showed, in and other work, different cytokine profiles produced by macrophages induced by lactobacilli, bifidobacteria, and bacteroides strains (21, 71). Similar observations were previously reported by Medina et al. (72). These authors demonstrated different abilities of *Bifidobacterium* strains to modulate *in vitro* production of cytokines by PBMCs, suggesting that they could drive immune responses in different directions. Maassen et al. (73) showed that different *Lactobacillus* strains induce distinct mucosal cytokine profiles and possess differential intrinsic adjuvant properties. These authors suggested that rational *Lactobacillus* strain selection provides a strategy to influence cytokines expression and thereby influence immune responses. Considering only our results of inflammatory profile in macrophages, strains such as CRL72, CRL1446, CRL352, CRL431, and CRL117 with a medium inflammatory profile could provide protection against the early stage of infection *via* Th1 with TNF-α and IL-6 production (74).

According to Jaedicke et al. (59), increased leptin concentrations in obesity may drive monocytes into a more activated, macrophage-like, pro-inflammatory state, possibly by affecting expression of TLRs and CD14, thereby enhancing LPS responsiveness and contributing to increased inflammation. Furthermore, knowing that leptin enhances T cell proliferation and Th1 pro-inflammatory cytokine production *in vitro* (31), we proceed to evaluate leptin and cytokines production by adipocytes in mono and coculture and the presence of a functional Ob-Rb (leptin receptor) in macrophages. Other authors showed that bacterial strains have different ability to modulate leptin secretion by adipocytes (26, 75, 76).

We evaluated whether Ob-Rb expression is regulated during macrophage activation by different LAB strains. Ob-Rb positive cells increased after LPS stimulation (positive control) and its expression diverged among different LAB stimuli. A previous study demonstrated that macrophages and B and T lymphocytes constitutively express low levels of Ob-Rb, and that this expression is upregulated, in terms of both percentage of positive cells and receptor density, in response to activation of these immune cells (31). We observed that “more inflammatory” strains produced more leptin as well as increased Ob-Rb receptor expression on macrophages, which is according to the notion of pro-inflammatory property of leptin and its adverse role in obesity (28).

Stimulating adipocytes/RAW264.7 cells with LAB results in marked upregulation of cytokine and leptin production in comparison with an adipocyte culture. Other authors, using the coculture system, showed inhibition of the cycle of stimulation of adipocytes and macrophages through the inhibition of macrophage-mediated pro-inflammatory cytokines and upregulation of adiponectin in adipocytes (77). These favorable effects may suppress chronic inflammation in adipose tissue. De Palma et al. also demonstrated, in another coculture system (PBMC and Caco cells), that some microorganisms had different behavior (cytokine production) using individual cell cultures (78). Furthermore, we observed significant production of IL-6 and MCP-1 in a coculture compared to the effect in macrophage cells; these two molecules are related to macrophage infiltration and insulin resistance in adipose tissue (40, 79). In addition, it is estimated that between 15 and 35% of the systemic IL-6 levels come from adipose tissue in obese individuals (79).

In our study, we used viable LAB strains and observed a modulation, strain dependent, on the production of leptin in adipocytes in mono- and coculture with macrophages. However, like other authors, we suggest that mediators produced by other cells in adipose tissue, such as macrophages, influence the secretory capacity of adipocytes and vice versa, because of the difference in the secretion of leptin, cytokines, and chemokine, comparing mono- and coculture (80, 81).

We observed that CRL431 strain showed a significant reduction in leptin levels compared to control (—), when adipocytes were stimulated, but significantly higher levels than LPS in macrophage–adipocyte–LAB coculture. A similar pattern occurred with strain CRL1434, which showed significantly higher leptin levels of LPS by stimulating adipocytes, but in the coculture system it presented values similar to LPS. This clearly reflects the intercommunication between these two cells (macrophages and adipocytes) and that LAB can affect the production of adipokines in this system.

In order to determine an association between the different strains, regarding their inflammatory profile, Ob-Rb receptor induction and their effect on leptin production, the PCA was carried out. In this study, CRL355, CRL431, CRL576, and CRL352 strains that induced high levels of leptin could be exploited in diseases with low concentrations of leptin. On the contrary, downregulation of circulating leptin levels may be considered as possible strategy to intervene on some inflammatory and autoimmune conditions (28). In the latter situation, strains such as CRL117, CRL350, CRL143 CRL575, CRL1446, and CRL66 could be useful. Miyazawa et al. demonstrated that increased pro-inflammatory cytokines (IL-6, IL-12, and TNF-α) inhibited the differentiation of a fibroblast cell line (3T3L1) in adipocytes, suggesting that acute inflammation stimulated by lactobacilli could be the mechanism by which adipogenic differentiation is suppressed, leading to adipocyte hypertrophy (33).

We suggest that the CRL575, CRL143, CRL117, CRL352, CRL576, CRL355, CRL431, and CRL1434 strains that induce high levels of leptin (either in adipocytes or in the Mac-Adi-BL system) could be oriented to the use in nutritional therapies for diseases that occur with reduced levels of leptin. In an opposite case, decreased levels of this adipokine would be necessary for the intervention of inflammatory or autoimmune pathologies (28), where CRL72, CRL66, and CRL1446 strains could be used.

It is noteworthy that CRL1446 strain induced a decline in leptin production. This strain has antioxidant, hypoglycemic, and hypocholesterolemic properties in animal studies (23, 82), thereby could be an interesting alternative for treating obesity, which is characterized by high leptin levels. CRL431 strain, which is a probiotic microorganism that demonstrated positive immunological effects in undernourished hosts (17, 38), was located, in the PCA analysis, next to strains that induced middle leptin production. Other authors showed the specific effect of LAB on the response of porcine adipocytes to TNF-α stimulation using conditioned media from LAB-stimulated intestinal immune cells (32). They suggested that lactobacilli may suppress differentiation of preadipocytes through macrophage activation and production of Th1 cytokines and could be effective in improving Th1 response not only in the gut but also in the systemic compartment as well. Then, our data show that different LAB strains could be capable of regulating adipokine expression in adipose tissue and suggest that they could have the potential to functionally modulate adipose inflammation when orally administered.

**Figure 6:**
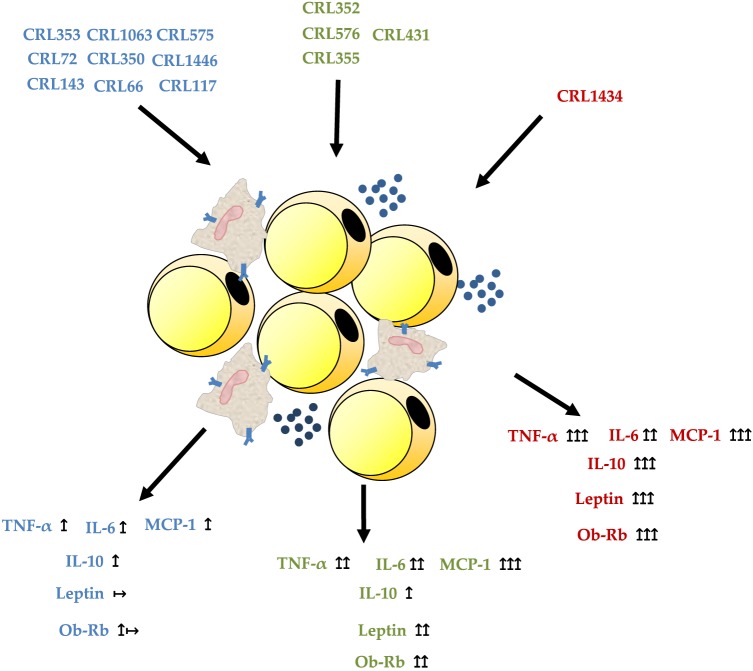
**Possible inmuno- and adipokine modulation of lactic acid bacteria in an *in vitro* macrophage/adipocyte system**.

We conclude that macrophage/adipocyte coculture is the most appropriate system for the selection of strains with the ability to modulate cytokine and leptin secretion. Our results showed that a stimulation of adipocyte monoculture with LAB strains exerts different effects than those observed in macrophage/adipocyte cocultures. This could be explained by the strong relationship between macrophages content and adipocytes in the adipose tissue, which boosts production of pro-inflammatory molecules and acute-phase proteins associated with obesity. Therefore, the regulation of TNF-α, IL-6, MCP-1, IL-10, and leptin and Ob-Rb receptor could be considered as biomarkers for immunobiotic strains. The use of microorganisms with low and middle inflammatory properties and ability to modulate leptin levels could be a strategy for the treatment of some metabolic diseases associated with immune abnormalities. In Figure 6, we show the possible inmuno- and adipokine modulation of LAB in an *in vitro* adipocyte system. This approach could be easily applicable in obesity and immune-deficient conditions like undernutrition. So, preclinical studies are currently being developed with the oral administration of CRL1446, CRL1434, and CRL431 (selected LAB in this work) in different experimental models of malnutrition (data no showed). The elucidation of the mechanisms involved in the modulation of adipose inflammation by LAB strains requires further studies associated with Ob-Rb and TLR expression.

## Author Contributions

Conceived and designed the experiments: EF and PG-C. Performed the experiments: EF, RR, and MCAM. Analyzed the data: EF, RR, MCAM, RM, and PG-C. Wrote draft and the final version of the manuscript: SG and PG-C.

## Conflict of Interest Statement

The authors declare that the research was conducted in the absence of any commercial or financial relationships that could be construed as a potential conflict of interest.
